# Tracking Japan’s development assistance for health, 2012–2016

**DOI:** 10.1186/s12992-020-00559-2

**Published:** 2020-04-15

**Authors:** Shuhei Nomura, Haruka Sakamoto, Maaya Kita Sugai, Haruyo Nakamura, Keiko Maruyama-Sakurai, Sangnim Lee, Aya Ishizuka, Kenji Shibuya

**Affiliations:** 1 Department of Global Health Policy, Graduate School of Medicine, The University of Tokyo, Tokyo, Japan; 2 Department of Health Policy and Management, School of Medicine, Keio University, Tokyo, Japan; 3 Institute for Global Health Policy Research (iGHP), Bureau of International Health Cooperation, National Center for Global Health and Medicine, Tokyo, Japan; 4 Department of International Affairs and Tropical Medicine, Tokyo Women’s Medical University, Tokyo, Japan; 5 Department of Clinical Epidemiology, Translational Medical Center, National Center of Neurology and Psychiatry, Tokyo, Japan; 6 Disease Control and Prevention Center, National Center for Global Health and Medicine, Tokyo, Japan; 7 Bureau of International Health Cooperation, National Center for Global Health and Medicine, Tokyo, Japan; 8 Department of Public Health, Graduate School of Medicine, Juntendo University, Tokyo, Japan; 9 School of Global Studies and Collaboration, Aoyama Gakuin University, Tokyo, Japan

**Keywords:** Development assistance for health, Health policy, Japan

## Abstract

**Background:**

Development assistance for health (DAH) is one of the most important means for Japan to promote diplomacy with developing countries and contribute to the international community. This study, for the first time, estimated the gross disbursement of Japan’s DAH from 2012 to 2016 and clarified its flows, including source, aid type, channel, target region, and target health focus area.

**Methods:**

Data on Japan Tracker, the first data platform of Japan’s DAH, were used. The DAH definition was based on the Organisation for Economic Co-operation and Development’s (OECD) sector classification. Regarding core funding to non-health-specific multilateral agencies, we estimated DAH and its flows based on the OECD methodology for calculating imputed multilateral official development assistance (ODA).

**Results:**

Japan’s DAH was estimated at 1472.94 (2012), 823.15 (2013), 832.06 (2014), 701.98 (2015), and 894.57 million USD (2016) in constant prices of 2016. Multilateral agencies received the largest DAH share of 44.96–57.01% in these periods, followed by bilateral grants (34.59–53.08%) and bilateral loans (1.96–15.04%). Ministry of Foreign Affairs (MOFA) was the largest contributors to the DAH (76.26–82.68%), followed by Ministry of Finance (MOF) (10.86–16.25%). Japan’s DAH was most heavily distributed in the African region with 41.64–53.48% share. The channel through which the most DAH went was Global Fund to Fight AIDS, Tuberculosis, and Malaria (20.04–34.89%). Between 2012 and 2016, approximately 70% was allocated to primary health care and the rest to health system strengthening.

**Conclusions:**

With many major high-level health related meetings ahead, coming years will play a powerful opportunity to reevaluate DAH and shape the future of DAH for Japan. We hope that the results of this study will enhance the social debate for and contribute to the implementation of Japan’s DAH with a more efficient and effective strategy.

## Background

Universal health coverage (UHC) is the cornerstone of sustainable and inclusive growth. The promotion of UHC to ensure that all people receive quality health services they need without financial hardship contributes to the development of human resources and security [1]. At the joint session of Finance and Health Ministers of the Group of 20 (G20) held in Osaka, Japan in June 2019, the “G20 Shared Understanding on the Importance of UHC Financing in Developing Countries” was confirmed, and agreement was reached to accelerate our global efforts to promote UHC through fair, equitable and preferential use of domestic resources and further invest in primary health care (PHC) services [2]. Furthermore, at the Seventh Tokyo International Conference on African Development (TICAD VII) held in Yokohama, Japan in August 2019, the Yokohama Declaration 2019 was adopted to promote a robust and sustainable society for human security in Africa region, including the achievement of the Sustainable Development Goals (SDGs) and African Union (AU) Agenda 2063 as well as UHC [3]. Prioritizing domestic resources and promoting private investment in Africa were also agreed as priority areas [3].

For many donors, development assistance for health (DAH) is one of the most important tools for promoting diplomacy and international cooperation with developing countries. For Japan, in 2013, the Ministry of Foreign Affairs (MOFA) issued the Global Health Diplomacy Strategy, officially positioning health as a pillar of government foreign diplomacy [4, 5]. As one of the concrete measures, the strategy aims to ensure human security by promoting UHC in developing countries using DAH [6]. Human security, as an universal definition, “protects the vital core of all human lives in ways that enhance human freedoms and human fulfillment” [7]. Through its contribution to health, the DAH helps build the human capital necessary for economic development [8], resulting in greater self-reliance of recipient countries [9]. Also, in an increasingly interconnected world, epidemics, antimicrobial resistance (AMR), and other health threats are easily spread, and efforts to prevent or suppress these threats in one country often benefit neighboring and distant countries [10]. The DAH therefore can provide a way for donors and recipient countries to benefit and share global prosperity [11].

Importantly, DAH functions as a support for issues that cannot be adequately addressed with domestic and private funds to achieve SDGs, including the SDG target 3.8 on UHC. In addition, there is an increasing need to act as a catalyst to strengthen capacity to mobilize and properly manage and disburse domestic and private funds [12]. DAH must also adapt to the growing health effects of climate change, conflict, and refugees/migrants crisis, and global political trends that emphasize national interests [13].

In early 2019, Japanese former Foreign Minister Mr. Taro Kono (until September 2019) stated that there was no guarantee that official development assistance (ODA) would increase in the future because of Japan’s fiscal deficit [14]. As stated in the government official documents and by Mr. Kono himself, there is a need for more efficient and effective implementation of ODA/DAH and greater transparency and accountability because ODA/DAH is funded by taxes [15, 16].

In Japan, decision-making and implementation of ODA is led by MOFA based on the Development Cooperation Charter, reflecting the wishes of other ministries and agencies, such as the Ministry of Finance (MOF) and Japan International Cooperation Agency (JICA: an implementation agency that coordinates bilateral ODA). In 1992, the ODA Charter was created by the Cabinet Office with Asia as a priority region, indicating the basic principles of supporting the economic growth and social development of developing countries through ODA [17]. In 2015, it was revised for the second time since the revision in 2003, and the name was changed to “Development Cooperation Charter” [18]. The core of this revised, new Charter is a deep commitment to a proactive contribution to peace standing on the concept of human security [5]. It also added measures that have not been considered within the framework of ODA, such as strengthening cooperation with private sectors; and clarified that ODA for military operations must be limited to non-military purposes, such as disaster relief. It puts emphasis not only on the interests of developing countries, but also on securing ‘national interests’, indicating Japan’s willingness to actively engage in international cooperation that would contribute to Japan’s security and economic growth. In the same year, MOFA also published the “Basic Design for Peace and Health (Global Health Cooperation)” as a guideline for global health policy under the new Charter. In this guideline, they put three areas as their priority: promotion of resilient global health governance able to respond to public health crises and natural disasters based on the concept of human security, including the realization of UHC; utilization of Japanese expertise, experience, medical products and technologies; and tailored support in response to diversification of regional needs [19].

In practice, however, due to its complex and fragmented administrative procedures and structures, the actual overall picture and flow of DAH has been unclear to date, and strategic decision-making and implementation across ministries and agencies are not sufficient. For example, there is no shared priority among ministries on their own commitments and no inter-ministry collaboration in the budget acquisition process [20]. JICA and other relevant organizations and domestic stakeholders also have their own policies, limiting strategic policy coordination with the government [20]. Here, we present, for the first time, an overview of Japan’s DAH, by examining the tracking of DAH using data on Japan Tracker, the first data platform of Japan’s DAH that the authors were in charge of [21]. The results of this study will contribute to an effective and strategic DAH decision-making and implementation across/within ministries and agencies.

## Methods

### Data

Data on ODA projects from 2012 to 2016 administered by MOFA were used. This data includes, for each project and year, gross disbursements of ODA, source (contributing ministry/agency), aid type (bilateral grant, including technical assistance; bilateral loan; earmarked funding to multilaterals [that is also called as ‘bi-multi’ and was reported as bilateral ODA]; and core funding to multilateral agencies [i.e. assessed contributions and non-earmarked funding]), target country/region, and target health focus area. Health focus area was based on purpose codes (also known as Creditor Reporting System [CRS] codes) for sector classification defined by Organisation for Economic Co-operation and Development’s (OECD) Development Assistance Committee (DAC) [22]. Purpose codes used for DAH were 120 (Health) and 130 (Population policies/Programs and reproductive health) based on the previous studies, including the 17 five-digit purpose codes [23–25]. The gross domestic product (GDP) deflator was used to convert current prices for 2012–2015 to constant prices at 2016 (131.34 for 2012, 107.06 for 2013, 100.43 for 2014, and 89.67 for 2015) [26].

### Imputed multilateral aid to health

Regarding core funding to non-health-specific multilateral agencies (e.g., World Bank), where it was not possible to directly identify DAH out of the ODA and its flows to target country/region and health focus area, they were estimated based on the OECD methodology for calculating imputed multilateral ODA as follows [27]. Step 1: based on reports from multilateral agencies to the OECD [28], ODA flows to the health sector of each agency (i.e., DAH) were calculated as a percentage of total ODA disbursements (α: health sector share of the agency’s total ODA). Step 2: based on this report [28], each agency’s DAH flows to each target country/region and each health focus area were calculated (β: target country/region-specific share of the agency’s DAH, and γ: health focus area-specific share of the agency’s DAH). Step 3: multiplying α, β, and γ obtained for each multilateral agency by the total ODA from Japan, we estimated flows of Japan’s DAH through the agency. For example, the MOF’s multilateral DAH through the World Bank was estimated by multiplying the total ODA from the MOF to the World Bank by α. In addition, MOF’s DAH through the World Bank to a particular target country/region and health focus area was estimated as total ODA × α × β and total ODA × α × γ, respectively.

### Primary health care and health system strengthening

In the spirit of both Alma-Ata and Astana, a well-functioning PHC system is regarded as the foundation for countries that successfully finance and provide quality health services to their entire population; this is essential to achieve UHC [29, 30]. Although the current CRS system does not facilitate standardized measurement of DAH for PHC, Shaw et al. (2015) attempted to define DAH on ‘PHC delivery’ versus on ‘health system strengthening (HSS)’ in support of PHC delivery, using CRS purpose code data, and our study followed their definition and methodology and estimated how much Japan’s DAH was invested in PHC and HSS [31]. In short, as in the previous study, our working definition of PHC focused only on inputs that are under the control of the health system itself, so intersectoral interventions (e.g., safe water, sanitation, and hygiene) were not considered. Our scope of PHC therefore included treatment of diseases and injuries, including the provision of essential medicines; reproductive health; prevention, detection and treatment of HIV/AIDS, tuberculosis and malaria; public health measures, preventive health care, promotion and education of healthy behavior, good nutrition, and immunization. In this study, we referred to DHA for such scope as being most relevant to ‘PHC delivery’.

Meanwhile, in order for PHC to function properly, system-wide investments are necessary: for example, effective priority setting system; sound management, administrative, financial, and technical capacities; adequate human resources and institution capacity; up-to-date health information systems for monitoring and evaluation of policies and programs; and appropriate regulatory, governance, finance, and accountability mechanisms. In this study, we referred to DAH for such investment as being most relevant to ‘HSS’ in support of PHC delivery, as in the previous study [31]. Our working definition of HSS is therefore much narrower than the extensive discussion of HSS often found in the literature, where most public expenditures aimed at improving health care can be interpreted as HSS. A discussion of their detailed definition and justification can be found in previous studies [31]. A list of corresponding CRS purpose codes for PHC and HSS can be found in the resulting table of this study.

## Results

Japan’s DAH was estimated at 1472.94 (2012), 823.15 (2013), 832.06 (2014), 701.98 (2015), and 894.57 million USD (2016) in constant prices of 2016. The gross disbursements of DAH by source and aid type are presented in Table 1. Except for in 2013, DAH contributed to multilateral agencies had the largest share of about 50% among all aid types. The share of bilateral (grants) was about 40% and that of bilateral (loans) was about 10% in the periods. MOFA accounted for majority of the DAH contribution (79.99%, 2016), with MOF (10.86%, 2016) and Ministry of Health, Labour and Welfare (MHLW) (9.13%, 2016) following in that order.

**Table 1:** Development assistance for health by source and type, 2012–2016 (2016 USD in million, %)

Source	Year	Bilateral (loans)	Bilateral (grants)	Multilateral	Total
MOFA	2012	84.04 (6.72)	582.99 (46.64)	507.14 (43.19)	1174.18
	2013	16.13 (2.57)	436.31 (69.50)	175.33 (27.93)	627.76
	2014	38.61 (5.71)	311.88 (46.13)	325.64 (48.16)	676.12
	2015	105.58 (18.19)	242.22 (41.73)	232.59 (40.07)	580.39
	2016	94.97 (13.27)	363.81 (50.84)	256.78 (35.88)	715.56
MHLW	2012	–	0.43 (0.55)	77.29 (99.45)	77.71
	2013	–	0.26 (0.42)	60.97 (99.58)	61.23
	2014	–	0.43 (0.85)	50.22 (99.15)	50.65
	2015	–	0.38 (0.92)	40.97 (99.08)	41.35
	2016	–	0.61 (0.75)	81.10 (99.25)	81.71
MOF	2012	–	–	219.9 (100)	219.90
	2013	–	–	133.8 (100)	133.80
	2014	–	–	98.47 (100)	98.47
	2015	–	–	80.05 (100)	80.05
	2016	–	–	97.14 (100)	97.14
Others	2012	–	0.45 (39.16)	0.7 (60.84)	1.15
	2013	–	0.36 (100)	–	0.36
	2014	–	6.80 (99.84)	0.01 (0.16)	6.81
	2015	–	0.18 (94.41)	0.01 (5.59)	0.20
	2016	–	0.15 (93.15)	0.01 (6.85)	0.16
All	2012	84.04 (5.71)	583.87 (39.64)	805.03 (54.65)	1472.94
	2013	16.13 (1.96)	436.92 (53.08)	370.1 (44.96)	823.15
	2014	38.61 (4.64)	319.11 (38.35)	474.34 (57.01)	832.06
	2015	105.58 (15.04)	242.79 (34.59)	353.62 (50.37)	701.98
	2016	94.97 (10.62)	364.58 (40.75)	435.02 (48.63)	894.57

*MOFA* Ministry of Foreign Affairs; *MHLW* Ministry of Health, Labour and Welfare; *MOF* Ministry of Finance. Others include Ministry of Agriculture, Forestry and Fisheries (MAFF); Ministry of Economy, Trade and Industry (METI); Ministry of Defense; Cabinet Office; and prefectures.

**Fig. 1:**
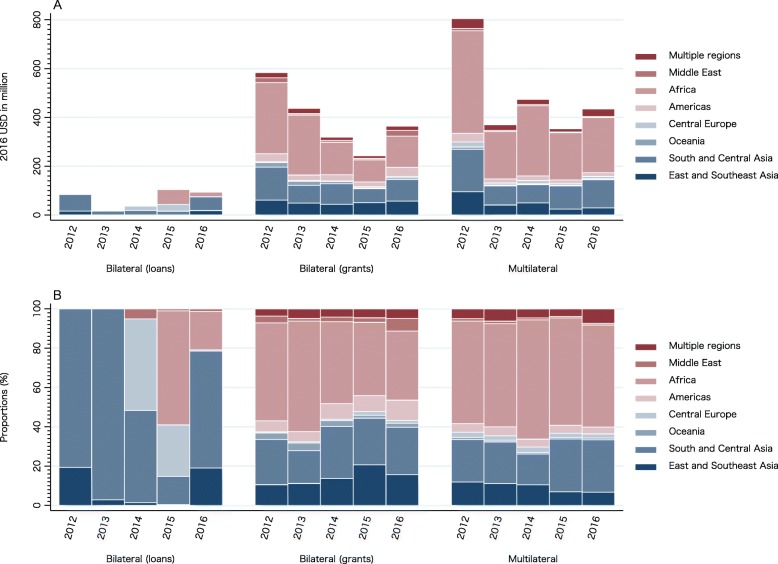
Development assistance for health by aid type and target region, 2012–2016: (A) value, (B) share

Japan’s DAH was most heavily distributed in the African region, with a range of 41.64–53.45% share between 2012 and 2016, followed by South and Central Asia (20.32–29.11%). Figure 1 shows Japan’s DAH by aid type and target region. In bilateral (grants) and multilateral, Japan’s DAH was allocated the most to Africa. As for bilateral (loans), the dominant focus was on South and Central Asia, with the exception of 2015. Exact values for Fig. 1 can be found in Supplementary Table 1.

**Fig. 2:**
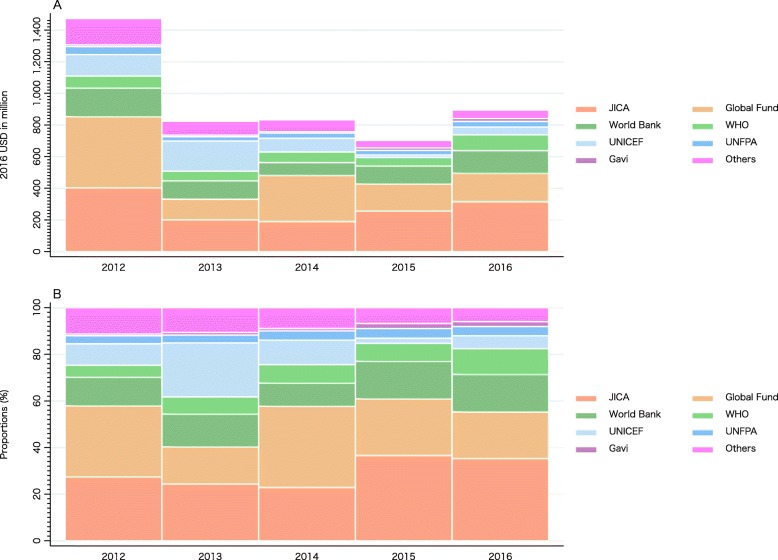
Development assistance for health by channels, 2012–2016: (A) value, (B) share WHO: World Health Organization; UNFPA: United Nations Population Fund; UNICEF: United Nations Children’s Fund; UNDP: United Nations Development Programme; Global Fund: The Global Fund to Fight AIDS, Tuberculosis and Malaria; Gavi: Gavi, The Vaccine Alliance; JICA: Japan International Cooperation Agency (Japan’s bilateral aid agency). Others include Joint United Nations Programme on HIV/AIDS (UNAIDS), Food and Agriculture Organization (FAO), United Nations Relief and Works Agency for Palestine Refugees in the Near East (UNRWA), World Food Programme (WFP), NGOs, etc

Figure 2 shows Japan’s DAH by major channel. The Global Fund to Fight AIDS, Tuberculosis and Malaria (Global Fund) (20.04–34.89%) or JICA (22.86–36.58%) channeled the most Japanese DAH between 2012 and 2016, followed by the World Bank (9.92–16.11%) or World Health Organization (WHO) (5.17–11.01%). Exact values for Fig. 2 can be found in Supplementary Table 2.

Supplementary Figure 1 shows the distribution of earmarked funding (bi-multi) and core funding to multilateral agencies for DAH in 2012–2016. The majority of DAH to United Nations Children’s Fund (UNICEF) was earmarked funding (83.69–100%), while only a portion of DAH to WHO and United Nations Population Fund (UNFPA) was earmarked funding: 3.45–30.00% and 3.86–46.15%, respectively. DAH to Gavi has also been mostly earmarked funding for the last two years. DAH to Joint United Nations Programme on HIV/AIDS (UNAIDS), United Nations Development Programme (UNDP), Global Funds, and development banks were core funding only. Exact values for Supplementary Figure 1 can be found in Supplementary Table 3.

**Fig. 3:**
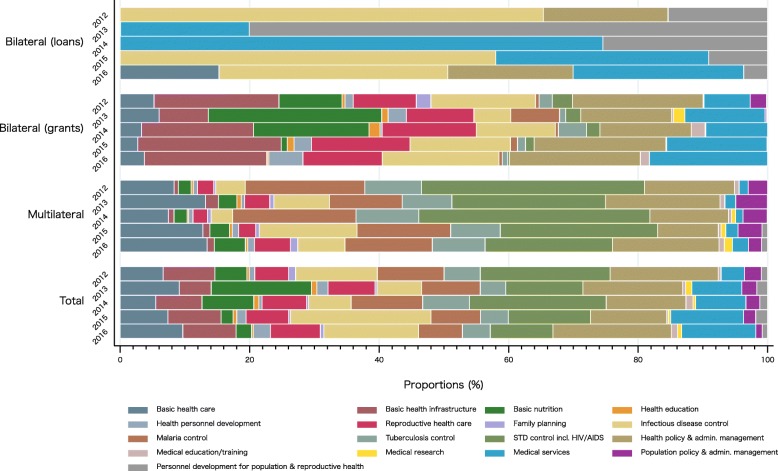
Development assistance for health by aid type and health focus area, 2012–2016 STD: sexually transmitted disease. CRS purpose code: Basic health care = 12220; Basic health infrastructure = 12230; Basic nutrition = 12240; Health education = 12261; Health personnel development = 12281; Reproductive health care = 13020; Family planning = 13030; Infectious disease control = 12250; Malaria control = 12262; Tuberculosis control = 12263; STD control including HIV/AIDS = 13040; Health policy and administrative management = 12110; Medical education/training = 12181; Medical research = 12182; Medical services = 12191; Population policy and administrative management = 13010; Personnel development for population and reproductive health = 13081

In Japan’s DAH, health policy and administrative management, medical services, infectious disease control, and sexually transmitted disease (STD) control including HIV/AIDS were the priority areas, which occupied a share over about 10% every year. However, by aid type the trend was different (Fig. 3). Infectious disease control had largest shares in bilateral (loans) except for 2013–2014, while STD control including HIV/AIDS had the largest share in multilateral. For bilateral (grants), on the other hand, Japanese funds also focused around basic health infrastructure and health policy and administrative management. Basic nutrition demonstrated a steep decline in its share in 2015–2016 among the bilateral grants. It was 26.78% in 2013, but fell to 0.08% in 2016. Exact values for Fig. 3 can be found in Supplementary Table 4.

**Fig. 4:**
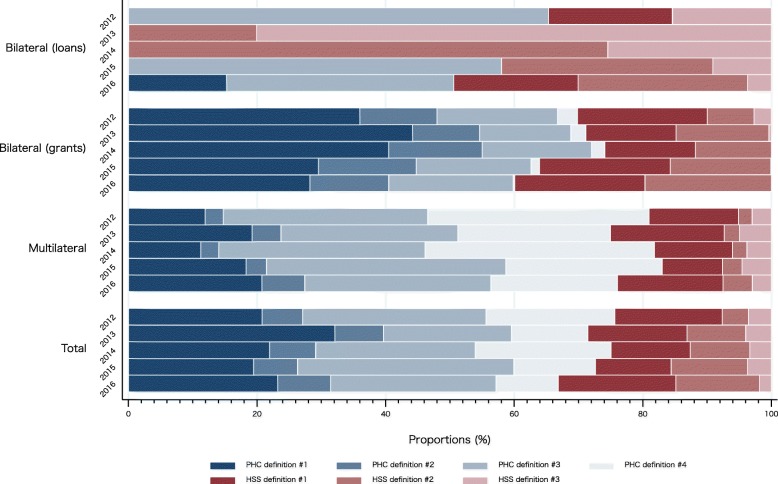
Development assistance for health to primary healthcare and health system strengthening by aid type, 2012–2016. PHC: public health care; HSS: health system strengthening. PHC definition #1 = Basic health care and infrastructure (CRS purpose codes: 12220, 12230, 12240, 12261, 12281); PHC definition #2 = Reproductive health care and family planning (13020, 13030); PHC definition #3 = Infectious disease control, including malaria and tuberculosis (12250, 12262, 12263); PHC definition #4 = Sexually transmitted disease (STD) control including HIV/AIDS (13040); HSS definition #1 = Health policy, administration & management (12110); HSS definition #2 = Medical services, training & research (12181, 12182, 12191); HSS definition #3 = Population policy & administration (13010, 13081)

To quantify levels and trends in DAH to PHC and HSS, the 17 five-digit purpose codes were grouped into two broad clusters (PHC and HSS). As reported in Fig. 4 with exact values presented in Supplementary Table 5, the DAH for PHC and HSS was then disaggregated into four and three narrow definitions of PHC and HSS, respectively. Between 2012 and 2016, 66.84–75.67% of DAH were allocated to PHC (broader PHC definition) and 24.33–33.16% to HSS (broader HSS definition). According to the PHC definition #1–4, 31.80–48.59% of DAH was allocated to infectious disease control (definition #3) and STDs (definition #4).

## Discussion

This study provided, for the first time, an estimated gross disbursement of Japan’s DAH and its flows. Japan’s DAH was found to be approximately 900 million USD in 2016. The main source of DAH was MOFA. According to the OECD statistics, Japan’s gross disbursements of ODA in 2016 amounted to 16.26 billion USD [28], which means that the share of DAH in ODA was about 5.5%.

In accordance with the ODA Charter, Japan has traditionally placed Asia, which has a close relationship with Japan, as a priority region [17]. Meanwhile, we revealed that approximately half of Japan’s bilateral and multilateral DAH were allocated to the African region in the study periods (Fig. 1). This finding may reflect Japan’s recent efforts to strengthen its diplomatic relations with African countries through various efforts including TICAD as well as Agenda 2063 and SDGs. For example, Japan hosted TICAD VI in Kenya in 2016 and launched the “UHC in Africa: Framework for Action” in partnership with the World Bank, WHO, the Global Fund, and the African Development Bank [32]. This is a roadmap for African countries to accelerate progress towards UHC and to monitor and assess their progress. In the same year, Japan hosted the Ise-Shima Group of Seven (G7) Summit, which was held in the aftermath of the Ebola crisis in Western Africa, providing an important opportunity for Japan to advance global health governance issues [33].

In addition, in May 2014, JICA signed, for the first time, an ODA loan agreement of up to 68.31 million USD (at current price in 2015) with the Government of the Federal Republic of Nigeria for the Polio eradication project [34]. This project aimed to contribute to the early eradication of polio in Nigeria by ensuring smooth vaccination of children under five years of age throughout the country through the procurement of polio vaccines. ODA loans to Africa in 2015 in Fig. 1 refer to this project.

This study examined the distribution of earmarked funding (bi-multi) and core funding to multilateral agencies for DAH in 2012–2016. Bi-multi funding is a resource to multilateral agencies over which the donor retains some degree of control on decisions regarding disposal of the funds. Such flows may be earmarked for a specific country, project, region, sector or theme. It is aid for bilateral functions channelled through multilateral agencies, and is therefore considered by the OECD and others as part of bilateral ODA [1–3].

On the other hand, core funding to multilateral agencies are used for a variety of purposes, some of which are channeled to global functions (e.g., provision of global public goods, management of cross-border externalities, and fostering of leadership and stewardship). Schäferhoff et al. (2015) estimated the total share of core funding going to global functions by agencies, as follows: WHO 62%, UNAIDS 40%, UNFPA 22%, UNICEF 12%, World Bank (International Development Association) 5%, Global Fund 10%, and Gavi 20% [23].

In Japan, core funding accounted for the majority of the DAH channeled through multilateral agencies, except for those through UNICEF and UNFPA. In particular, Japan’s core funding to WHO, which primarily focuses on global functions, has ranked 2nd in the world after the United States [35]. It may be said that Japan’s global functions in relation to the global trends is relatively high. This finding may be consistent with the direction of the Basic Design for Peace and Health, which emphasizes the strengthening of global functions based on the concept of human security. For example, at the Ise-Shima G7 Summit, Japan emphasized the promotion of aid for global functions both in the G7 Ise-Shima Leaders’ Declaration and G7 Ise-Shima Vision for Global Health [36, 37].

While the effective DAH allocation has long been discussed, it might be guided by a number of factors, including historical and traditional diplomatic relations, geographic proximity, strategic reciprocity, and trade-related considerations, particularly in bilateral aid; and not necessarily aligned with disease priorities for health aid in recipient countries and cost-effectiveness of interventions [38].

Globally, however, DAH growth has been stagnant over the past 10 years and limited financial resources are a universal constraint [39]; Japan is not exception. It is, therefore, an urgent policy issue to implement DAH strategies wisely, efficiently and effectively, while ensuring transparency.

### Synergies through the human security approach

Both in the Global Health Diplomacy Strategy and the Basic Design for Peace and Health, protecting human security has been a core concept of Japanese foreign policy [4, 5]. Human security is at a convergence that combines the competing policy issues that could threaten vital core of all human lives, including infectious disease epidemics (as exemplified by the recent 2014 Ebola outbreak [10] or pandemic influenza) as well as refugee and migration crises and climate change. Human security approach thus enriches the synergy between measures to address these issues. For example, among the nearly one million Rohingyas, an Islamic minority group, living in a refugee camp in Bangladesh, there is a growing concern about a serious infectious disease epidemic, including measles, cholera, and typhoid [40]. Also, as global warming progresses, the distribution of vectors such as mosquitoes that transmit Japanese encephalitis, dengue fever, malaria, and yellow fever, may expand [41, 42]. Human security approach will also contribute to the achievement of SDGs as well as AU Agenda 2063 by building a healthy, sustainable, and stable society. An important issue in the DAH strategy for donors is therefore to consider how donors should fund their human security efforts from a limited ODA budget, and in particular what is the optimal role of DAH in this context.

For example, Japan is one of the founding partners of the Global Fund and a major donor who contributed 20.04–34.89% of DAH to the Global Fund in 2012–2016. Since its establishment in 2002, an accumulated 3.46 billion USD has been contributed from Japan [43]. At the meeting of the Sustainable Development Goals Promotion Headquarters on June 2019, Prime Minister Shinzo Abe announced Japan’s new pledge of 840 million USD to the Global Fund’s Sixth Replenishment [44]. Infectious disease control is an important DAH strategy of Japan, which covered 19.89–33.64% of Japan’s DAH shares overall between 2012 and 2016, and was mostly channeled through the Global Fund. In the context of human security approach to climate change and refugees and migrants crisis, further scale-up of DAH investments in effective infectious disease control is expected.

Note that human security approach in this context means supporting people-centered, comprehensive, context-specific, and prevention-oriented responses that strengthen the protection and empowerment of all people, adopting partnerships across sectors, developing context-sensitive solutions, and supporting the realization of a world without fear, want, and dignity [45]. Caution is needed that while human security as a rationale for linking foreign policy and health introduces significant political power, sufficient attention must be paid to the possibility that national security interests may be skewed towards health and humanitarian issues [46]. It should also be noted that treating global health issues as national security threats, rather than universal issues to be concerned with the humanity, may cause an excessive concern surrounding diseases surveillance and a divide between affected countries and non-affected countries. In the past, for example, securitization was misused as a rationale for implementing HIV-based travel, migration, and immigration control policies and laws prohibiting the entry of people living with HIV [47].

### Health system strengthening for non-communicable diseases

The results also showed that between 2012 and 2016, approximately 70% of DAH were allocated to PHC, and remaining 30% to HSS. Although there are no established norms or benchmarks on the balance between PHC and HSS allocations in DAH, the High Level Task Force on Innovative International Financing for Health Systems (HLTF) proposed that approximately 15–26% of the additional resources would be required for HSS—that are broadly consistent with the above definitions—in order to achieve the Millennium Development Goals (MDGs) for low-income countries [48]. In terms of the MDGs, therefore, the balance between PHC and HSS in Japan’s DAH could be roughly reasonable. However, in today’s era of SDGs, the growing emphasis on social determinants of health makes it even more crucial that DAH strengthens health system, including institutional capacity (effectiveness of surveillance systems and laboratory networks, etc.), administrative and financial systems, and human resources development [49].

Donor-recipient countries face the challenges posed by health transition, i.e., a double burden of morbidity, mortality, and associated health care costs from increasing non-communicable diseases (NCDs) and continuing high communicable diseases [50]. PHC has played a successful role in the delivery of prevention and care interventions for communicable diseases, such as malaria, tuberculosis, and HIV/AIDS. However, it is imperative to expand the delivery of PHC in countries undergoing health transition in terms of health promotion and disease prevention and treatment in response to NCDs [51]. With limited resources, several studies suggested the need to take a diagonal approach of HSS to address NCDs, rather than disease-specific, vertical programs [52, 53]. HSS has the potential to improve the delivery of PHC in a cost-effective manner by dealing with the wide range of health problems encountered in health transition. HSS are emerging important focus of some multilateral agencies, such as the World Bank and the Global Fund (Japan’s major DAH channels), as well as the Gavi, The Vaccine Alliance.

There is an increasing debate as to why donor countries, including Japan, should invest more in NCDs [54]. A 2019 study demonstrated that recently only 1% or less of Japan’s DAH went to NCDs [39], whereas NCDs accounted for 40–50% of total disease burden in low- and lower-middle-income countries (LMICs) [55]. However, this does not imply that funds for infectious disease control should be used to scale-up to confront NCDs through HSS. Between 2012 and 2016, 31.80–48.59% of Japan’s DAH went to infectious diseases control including HIV/AIDS, which has aligned with disease burden in LMICs to some extent (or lower), where infectious disease accounted for about 40–50% of the total disease burden in LMICs [55]. Importantly, Japan’s DAH allocation should take full account of the health transition of DAH-recipient countries and make the burden of disease an important criterion for prioritizing resource allocation [12]. In the future, it will become increasingly important to promote prevention as well as treatment by focusing on HSS in recipient countries.

### Approach to support domestic resources and private investment

More effective health spending is needed in developing countries, and they should use all available resources. Recognizing this need, SDG 17 aims to strengthen domestic resource mobilization and improve domestic fiscal capacity for tax and other revenue collection [56]. In addition, under the 2015 Addis Ababa Action Agenda, countries pledged to achieve the SDGs, largely using domestic resources [57]. These are also recognized as common understanding to achieve UHC at the joint session of Finance and Health Ministers at the Osaka G20 Summit this year as well as at TIVAD VII [2, 3].

On the other hand, a recent study estimated that achieving UHC would require an increase in annual per capita health spending of more than 100 USD by 2030 in LMICs [58]. More spending may be needed, especially as the country develops economically and prices rise. This figure is much larger than DAH alone can cover. While taking into account the country’s own priorities, it is the most important strategic challenge for donors to consider how DAH can support the use and mobilization of domestic resources and how it can intervene in ways that reduce investment risks for the private sector [12].

For multilateral aid, the Global Financing Facility (GFF) and Global Action Plan for Healthy Lives and Well-Being for All is a new approach that leverages domestic resources as well as ongoing funding from private and public sources. Japan is one of the 10 donor countries of GFF as of May 2019. The first commitment, a pledge of 50 million USD, to the GFF by the Government of Japan was announced at the UHC Forum 2017 [59].

In addition, donors should be aware of the potential for the implementation of DAH to impair the ability of DAH-recipient countries to properly plan health budget disbursements, and should seek ways to avoid it. A 2016 study by the World Bank and other institutions found that the costs of using parallel systems of DAH and domestic resources were more than four times higher than relying solely on national financial systems and skills transfer [60]. Also, there is an evidence of negative correlation between DAH and domestic resources; DAH may constrain the domestic health budget and cause its significant portion substituted out of the health sector [61, 62]. Decision-making and implementation of DAH should consider how financial flows in DAH-recipient countries interact with each other.

### Limitation

While ODA system is well-known, many complexities are involved in its use. This study made use of DAH on gross disbursements rather than commitments as disbursements are actual distributions of committed aid funds, while the commitments are amount the donor agreed to make available to. In some cases, disbursements could be more volatile than commitments, conditional on specific country events (e.g., political instability), and absorptive capacity during any one year [63].

As noted in the previous study [31], it is difficult to draw a strong conclusion about the share of PHC and HSS for several reasons. First, there is a lack of global agreement on measurable indications for PHC and HSS. It also includes the lack of normative descriptions of the share of DAH by donors for PHC and HSS. The method developed in the previous study (and used in this study) can be reproduced using OECD/CRS data and may serve as a useful method to track future donor resources allocated to PHC/HSS.

Our estimates of DAH are not necessarily comparable to those of the Institute for Health Metrics and Evaluation (IHME) at the University of Washington, which also provides an alternative source of data on DAH [39]. IHME uses rather complicated mathematical procedures to classify aid based on a ‘word search’ of project/program content, rather than the long-established coding procedures followed by donors for the OECD/CRS data base. IHME estimates tend to be relatively large in value than our estimates based on the OECD coding procedures. For example, in Japan, a DAH of 2016 was estimated to be 895 million USD in this study, while IHME estimates was 1100 million USD (at constant price in 2018) [39]. This may be because the IHME’s estimation method using word search allows some consideration even in areas, such as ‘agriculture’ (CRS code 310), ‘water and sanitation’ (140), and ‘education’ (110) that the OECD coding procedures based on the CRS code does not consider as ‘health’ (120 + 130). In addition, in the OECD coding, there are 17 focus areas in the field of health, while IHME classifies health into 7 focus areas. IHME also provides very important data, although the methods of estimation and classification are different. However, this study adopted the OECD coding procedures, whose categorization is more familiar and straightforward for policy makers and government officials in Japan to understand.

This study used only data from Japan over a five-year period, and therefore does not provide long-term trends of DAH or comparisons with other countries, or any consideration from the perspective of Japan’s relative position in global health diplomacy. This is our next research scope.

## Conclusions

With many major high-level health related meetings ahead, coming years will play a powerful role in reevaluating DAH and shaping the future of DAH for the world and Japan. We hope that the results of this study, which provide an overview of DAH in Japan, will enhance the social debate for and contribute to the implementation of Japan’s DAH in a more efficient and effective strategy.

## Supplementary information

**Additional file 1: Figure S1.** Developing assistance for health channeled through multilateral agencies, 2012–2016. WHO: World Health Organization; UNAIDS: Joint United Nations Programme on HIV/AIDS; UNFPA: United Nations Population Fund; UNICEF: United Nations Children’s Fund; UNDP: United Nations Development Programme; AfDB: African Development Bank; AsDB: Asian Development Bank; IADB: Inter-American Development Bank; Global Fund: The Global Fund to Fight AIDS, Tuberculosis and Malaria; Gavi: Gavi, The Vaccine Alliance.

**Additional file 2: Table S1.** Development assistance for health by target region, 2012–2016 (2016 USD in million, %): (A) bilateral (loans), (B) bilateral (grants), (C) multilateral, (D) total. WHO: World Health Organization; UNAIDS: Joint United Nations Programme on HIV/AIDS; UNFPA: United Nations Population Fund; UNICEF: United Nations Children’s Fund; UNDP: United Nations Development Programme; AfDB: African Development Bank; AsDB: Asian Development Bank; IADB: Inter-American Development Bank; Global Fund: The Global Fund to Fight AIDS, Tuberculosis and Malaria; Gavi: Gavi, The Vaccine Alliance; JICA: Japan International Cooperation Agency. Other UN agencies include Food and Agriculture Organization (FAO), United Nations Relief and Works Agency for Palestine Refugees in the Near East (UNRWA), World Food Programme (WFP), etc. NGOs include International Planned Parenthood Federation, etc. Others include Global Environment Facility (GEF), etc. **Table S2.** Development assistance for health by channel, 2012–2016 (2016 USD in million, %). WHO: World Health Organization; UNAIDS: Joint United Nations Programme on HIV/AIDS; UNFPA: United Nations Population Fund; UNICEF: United Nations Children's Fund; UNDP: United Nations Development Programme; AfDB: African Development Bank; AsDB: Asian Development Bank; IADB: Inter-American Development Bank; Global Fund: The Global Fund to Fight AIDS, Tuberculosis and Malaria; Gavi: Gavi, The Vaccine Alliance; JICA: Japan International Cooperation Agency. Other UN agencies include Food and Agriculture Organization (FAO), United Nations Relief and Works Agency for Palestine Refugees in the Near East (UNRWA), World Food Programme (WFP), etc. NGOs include International Planned Parenthood Federation, etc. Others include Global Environment Facility (GEF), etc. **Table S3.** Developing assistance for health channeled through multilateral agencies, 2012–2016 (2016 USD in million, %). WHO: World Health Organization; UNAIDS: Joint United Nations Programme on HIV/AIDS; UNFPA: United Nations Population Fund; UNICEF: United Nations Children’s Fund; UNDP: United Nations Development Programme; AfDB: African Development Bank; AsDB: Asian Development Bank; IADB: Inter-American Development Bank; Global Fund: The Global Fund to Fight AIDS, Tuberculosis and Malaria; Gavi: Gavi, The Vaccine Alliance; JICA: Japan International Cooperation Agency. Other UN agencies include Food and Agriculture Organization (FAO), United Nations Relief and Works Agency for Palestine Refugees in the Near East (UNRWA), World Food Programme (WFP), etc. **Table S4.** Development assistance for health by health focus area, 2012–2016 (2016 USD in million, %): (A) bilateral (loans), (B) bilateral (grants), (C) multilateral, (D) total. STD: sexually transmitted disease. CRS purpose code: Basic health care = 12220; Basic health infrastructure = 12230; Basic nutrition = 12240; Health education = 12261; Health personnel development = 12281; Reproductive health care = 13020; Family planning = 13030; Infectious disease control = 12250; Malaria control = 12262; Tuberculosis control = 12263; STD control including HIV/AIDS = 13040; Health policy and administrative management = 12110; Medical education/training = 12181; Medical research = 12182; Medical services = 12191; Population policy and administrative management = 13010; Personnel development for population and reproductive health = 13081. **Table S5.** Development assistance for health for primary healthcare and health system strengthening, 2012–2016 (2016 USD in million, %): (A) bilateral (loans), (B) bilateral (grants), (C) multilateral, (D) total. PHC: public health care; HSS: health system strengthening. PHC definition #1 = Basic health care and infrastructure (CRS purpose codes: 12220, 12230, 12240, 12261, 12281); PHC definition #2 = Reproductive health care and family planning (13020, 13030); PHC definition #3 = Infectious disease control, including malaria and tuberculosis (12250, 12262, 12263); PHC definition #4 = Sexually transmitted disease (STD) control including HIV/AIDS (13040); Broader PHC definition = Definition #1 + #2 + #3 + #4; HSS definition #1 = Health policy, administration & management (12110); HSS definition #2 = Medical services, training & research (12181, 12182, 12191); HSS definition #3 = Population policy & administration (13010, 13081); Broader HSS definition = Definition #1 + #2 + #3.

## Data Availability

All data generated or analysed during this study are included in this published article and its supplementary information files.

## Abbreviations

AMR: Antimicrobial resistance
AU: African Union
CRS: Creditor reporting system
DAC: Development Assistance Committee
DAH: Development assistance for health
G20: Group of 20
GDP: Gross domestic product
GFF: Global Financing Facility
Global Fund: Global Fund to Fight AIDS, Tuberculosis and Malaria
HSS: Health system strengthening
IHME: Institute for Health Metrics and Evaluation
JICA: Japan International Cooperation Agency
LMICs: Low- and lower-middle-income countries
MAFF: Ministry of Agriculture, Forestry and Fisheries
METI: Ministry of Economy, Trade and Industry
MHLW: Ministry of Health, Labour and Welfare
MOF: Ministry of Finance
MOFA: Ministry of Foreign Affairs
NCDs: Non-communicable diseases
ODA: Official development assistance
OECD: Organisation for Economic Co-operation and Development
PHC: Primary health care
SDGs: Sustainable Development Goals
STD: Sexually transmitted disease
TICAD: Tokyo International Conference on African Development
UHC: Universal health coverage
WHO: World Health Organization
