# Correction to: Tracking Japan’s development assistance for health, 2012–2016

**DOI:** 10.1186/s12992-020-00640-w

**Published:** 2021-01-04

**Authors:** Shuhei Nomura, Haruka Sakamoto, Maaya Kita Sugai, Haruyo Nakamura, Keiko Maruyama-Sakurai, Sangnim Lee, Aya Ishizuka, Kenji Shibuya

**Affiliations:** 1 Department of Global Health Policy, Graduate School of Medicine, The University of Tokyo, Tokyo, Japan; 2 Department of Health Policy and Management, School of Medicine, Keio University, Tokyo, Japan; 3 Institute for Global Health Policy Research (iGHP), Bureau of International Health Cooperation, National Center for Global Health and Medicine, Tokyo, Japan; 4 Department of International Affairs and Tropical Medicine, Tokyo Women’s Medical University, Tokyo, Japan; 5 Department of Clinical Epidemiology, Translational Medical Center, National Center of Neurology and Psychiatry, Tokyo, Japan; 6 Disease Control and Prevention Center, National Center for Global Health and Medicine, Tokyo, Japan; 7 Bureau of International Health Cooperation, National Center for Global Health and Medicine, Tokyo, Japan; 8 Department of Public Health, Graduate School of Medicine, Juntendo University, Tokyo, Japan; 9 School of Global Studies and Collaboration, Aoyama Gakuin University, Tokyo, Japan

**Correction to: Glob Health (2020) 16: 32**

**https://doi.org/10.1186/s12992-020-00559-2**

Following publication of the original article [1], the authors reported a conversion error that concerned the estimated amounts of development assistance for health (DAH) for 2012–2015; when the authors converted the current prices of 2012–2015 to the constant prices of 2016 using the gross domestic product (GDP) deflator, they erroneously multiplied the current prices by the GDP deflator instead of dividing them.

Please find the details of this error in this correction.

Firstly, the ‘Results’ in the article’s Abstract stated that “Japan’s DAH was estimated at 1,472.94 (2012), 823.15 (2013), 832.06 (2014), 701.98 (2015), and 894.57 million USD (2016) in constant prices of 2016”, while it should state that “Japan’s DAH was estimated at 853.87 (2012), 718.16 (2013), 824.95 (2014), 873.04 (2015), and 894.57 million USD (2016) in constant prices of 2016”.

Secondly, the first sentence of the article’s Results section stated that “Japan’s DAH was estimated at 1,472.94 (2012), 823.15 (2013), 832.06 (2014), 701.98 (2015), and 894.57 million USD (2016) in constant prices of 2016”, while it should state that “Japan’s DAH was estimated at 853.87 (2012), 718.16 (2013), 824.95 (2014), 873.04 (2015), and 894.57 million USD (2016) in constant prices of 2016”.

In addition to the above mentioned parts of the article, the conversion error affected Table 1, Fig. 1a, Fig. 2a, and Additional file 2, for the data of 2012–2015; please find (the corrected version of) these files in this correction.

**Table 1:** Development assistance for health by source and type, 2012–2016 (2016 USD in million, %)

Source	Year	Bilateral (loans)	Bilateral (grants)	Multilateral	Total
MOFA	2012	48.72 (7.16)	337.96 (49.65)	293.99 (43.19)	680.67
	2013	14.07 (2.57)	380.66 (69.50)	152.97 (27.93)	547.70
	2014	38.28 (5.71)	309.21 (46.13)	322.85 (48.16)	670.34
	2015	131.31 (18.19)	301.25 (41.73)	289.26 (40.07)	721.81
	2016	94.97 (13.27)	363.81 (50.84)	256.78 (35.88)	715.56
MHLW	2012	–	0.25 (0.55)	44.80 (99.45)	40.05
	2013	–	0.23 (0.42)	53.19 (99.58)	53.42
	2014	–	0.43 (0.85)	49.80 (99.15)	50.22
	2015	–	0.47 (0.92)	50.95 (99.08)	51.43
	2016	–	0.61 (0.75)	81.10 (99.25)	81.71
MOF	2012	–	–	127.48 (100)	127.48
	2013	–	–	116.74 (100)	116.74
	2014	–	–	97.63 (100)	97.63
	2015	–	–	99.56 (100)	99.56
	2016	–	–	97.14 (100)	97.14
Others	2012	–	0.26 (39.16)	0.40 (60.84)	0.67
	2013	–	0.31 (100)	–	0.31
	2014	–	6.75 (99.84)	0.01 (0.16)	6.76
	2015	–	0.23 (94.41)	0.01 (5.59)	0.24
	2016	–	0.15 (93.15)	0.01 (6.85)	0.16
All	2012	48.72 (5.71)	338.47 (39.64)	466.68 (54.65)	853.87
	2013	14.07 (1.96)	381.20 (53.08)	322.89 (44.96)	718.16
	2014	38.28 (4.64)	316.38 (38.35)	470.29 (57.01)	824.95
	2015	131.31 (15.04)	301.95 (34.59)	439.78 (50.37)	873.04
	2016	94.97 (10.62)	364.58 (40.75)	435.02 (48.63)	894.57

*MOFA* Ministry of Foreign Affairs; *MHLW* Ministry of Health, Labour and Welfare; *MOF* Ministry of Finance. Others include Ministry of Agriculture, Forestry and Fisheries (MAFF); Ministry of Economy, Trade and Industry (METI); Ministry of Defense; Cabinet Office; and prefectures.

**Fig. 1:**
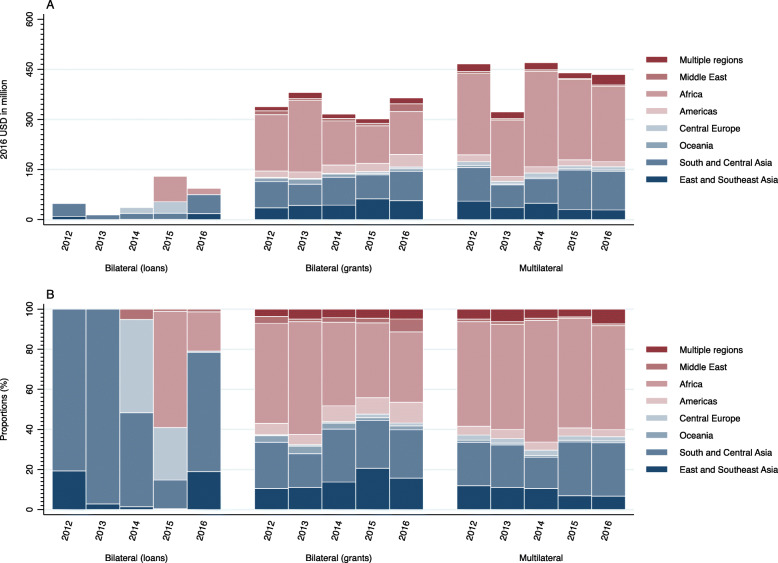
Development assistance for health by aid type and target region, 2012–2016: **a** value, **b** share

**Fig. 2:**
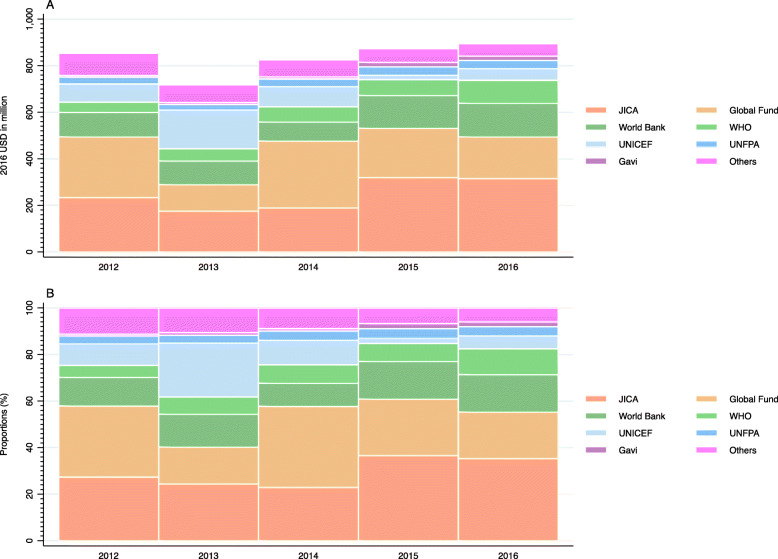
Development assistance for health by channels, 2012–2016: **a** value, **b** share WHO: World Health Organization; UNFPA: United Nations Population Fund; UNICEF: United Nations Children’s Fund; UNDP: United Nations Development Programme; Global Fund: The Global Fund to Fight AIDS, Tuberculosis and Malaria; Gavi: Gavi, The Vaccine Alliance; JICA: Japan International Cooperation Agency (Japan’s bilateral aid agency). Others include Joint United Nations Programme on HIV/AIDS (UNAIDS), Food and Agriculture Organization (FAO), United Nations Relief and Works Agency for Palestine Refugees in the Near East (UNRWA), World Food Programme (WFP), NGOs, etc

The errors have now been corrected in the original article.

Furthermore, the authors would like to assure the reader that the discussions proposed in their article were based on the part of the results not related to the conversion by the GBD deflator (i.e. percentage value rather than amount) and, therefore, that the miscalculated amounts of DAH mentioned above do not affect the interpretation or conclusions of the study.

The authors thank you for reading this correction, and apologize for any inconvenience caused.

## Supplementary Information

**Additional file 2:** **Supplementary Table 1.** Development assistance for health by target region, 2012–2016 (2016 USD in million, %): (A) bilateral (loans), (B) bilateral (grants), (C) multilateral, (D) total. **Supplementary Table 2.** Development assistance for health by channel, 2012–2016 (2016 USD in million, %). **Supplementary Table 3.** Developing assistance for health channeled through multilateral agencies, 2012–2016 (2016 USD in million, %). **Supplementary Table 4.** Development assistance for health by health focus area, 2012–2016 (2016 USD in million, %): (A) bilateral (loans), (B) bilateral (grants), (C) multilateral, (D) total. **Supplementary Table 5.** Development assistance for health for primary healthcare and health system strengthening, 2012–2016 (2016 USD in million, %): (A) bilateral (loans), (B) bilateral (grants), (C) multilateral, (D) total.
