# Supplementary figures and images for: Tracking Japan’s development assistance for health, 2012–2016
Source: Global Health. 2020 Apr 15;16:32. doi: 10.1186/s12992-020-00559-2 (PMC7161223; doi:10.1186/s12992-020-00559-2)

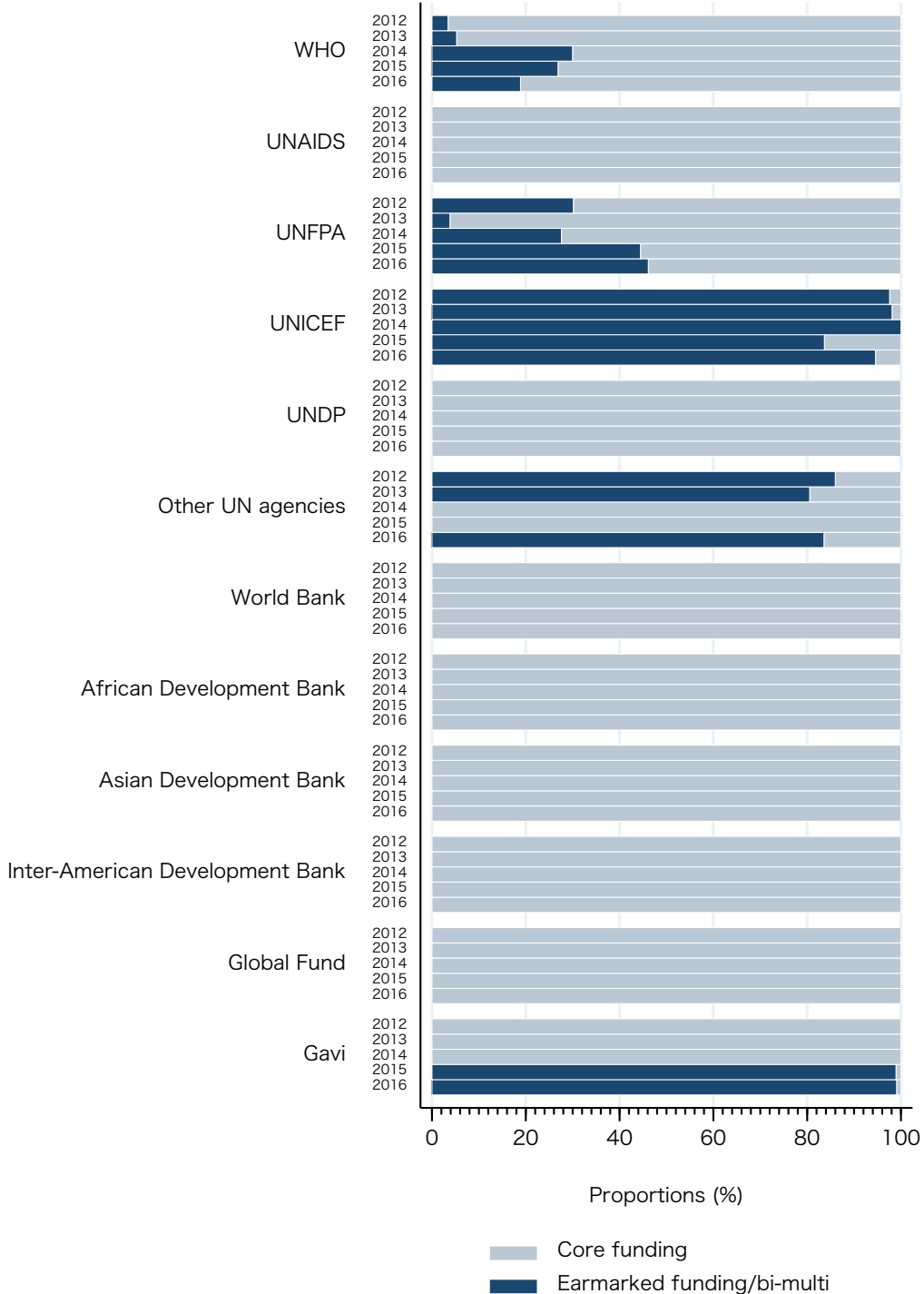

Supplement: Supplementary file 1 — Additional file 1: Figure S1. Developing assistance for health channeled through multilateral agencies, 2012–2016. WHO: World Health Organization; UNAIDS: Joint United Nations Programme on HIV/AIDS; UNFPA: United Nations Population Fund; UNICEF: United Nations Children’s Fund; UNDP: United Nations Development Programme; AfDB: African Development Bank; AsDB: Asian Development Bank; IADB: Inter-American Development Bank; Global Fund: The Global Fund to Fight AIDS, Tuberculosis and Malaria; Gavi: Gavi, The Vaccine Alliance. [file 12992_2020_559_MOESM1_ESM.pdf]
