# Supplementary material for: Tracking Japan’s development assistance for health, 2012–2016
Source: Global Health. 2020 Apr 15;16:32. doi: 10.1186/s12992-020-00559-2 (PMC7161223; doi:10.1186/s12992-020-00559-2)
Supplement: Supplementary file 2 — Additional file 2: Table S1. Development assistance for health by target region, 2012–2016 (2016 USD in million, %): (A) bilateral (loans), (B) bilateral (grants), (C) multilateral, (D) total. WHO: World Health Organization; UNAIDS: Joint United Nations Programme on HIV/AIDS; UNFPA: United Nations Population Fund; UNICEF: United Nations Children’s Fund; UNDP: United Nations Development Programme; AfDB: African Development Bank; AsDB: Asian Development Bank; IADB: Inter-American Development Bank; Global Fund: The Global Fund to Fight AIDS, Tuberculosis and Malaria; Gavi: Gavi, The Vaccine Alliance; JICA: Japan International Cooperation Agency. Other UN agencies include Food and Agriculture Organization (FAO), United Nations Relief and Works Agency for Palestine Refugees in the Near East (UNRWA), World Food Programme (WFP), etc. NGOs include International Planned Parenthood Federation, etc. Others include Global Environment Facility (GEF), etc. Table S2. Development assistance for health by channel, 2012–2016 (2016 USD in million, %). WHO: World Health Organization; UNAIDS: Joint United Nations Programme on HIV/AIDS; UNFPA: United Nations Population Fund; UNICEF: United Nations Children's Fund; UNDP: United Nations Development Programme; AfDB: African Development Bank; AsDB: Asian Development Bank; IADB: Inter-American Development Bank; Global Fund: The Global Fund to Fight AIDS, Tuberculosis and Malaria; Gavi: Gavi, The Vaccine Alliance; JICA: Japan International Cooperation Agency. Other UN agencies include Food and Agriculture Organization (FAO), United Nations Relief and Works Agency for Palestine Refugees in the Near East (UNRWA), World Food Programme (WFP), etc. NGOs include International Planned Parenthood Federation, etc. Others include Global Environment Facility (GEF), etc. Table S3. Developing assistance for health channeled through multilateral agencies, 2012–2016 (2016 USD in million, %). WHO: World Health Organization; UNAIDS: Joint United Nations Progra [file 12992_2020_559_MOESM2_ESM.docx]

**Supplementary table 1: Development assistance for health by target region, 2012–2016 (2016 USD in million, %): (A) bilateral (loans), (B) bilateral (grants), (C) multilateral, (D) total**

| A. bilateral (loans) |  |  |  |  |  |
| --- | --- | --- | --- | --- | --- |
| Target region | 2012 | 2013 | 2014 | 2015 | 2016 |
| East and Southeast Asia | 9.39 (19.28) | 0.39 (2.78) | 0.54 (1.40) | 0.52 (0.39) | 18.01 (18.96) |
| South and Central Asia | 39.33 (80.72) | 13.68 (97.22) | 17.96 (46.93) | 18.84 (14.34) | 56.55 (59.55) |
| Oceania | – | – | – | – | – |
| Central Europe | – | – | 17.81 (46.54) | 34.39 (26.19) | 0.60 (0.63) |
| Americas | – | – | – | – | – |
| Africa | – | – | – | 76.18 (58.02) | 18.53 (19.52) |
| Middle East | – | – | 1.96 (5.13) | 1.39 (1.06) | 1.28 (1.34) |
| Multiple regions | – | – | – | – | – |
| Total | 48.72 | 14.07 | 38.28 | 131.31 | 94.97 |
|  |  |  |  |  |  |
| B. bilateral (grants) |  |  |  |  |  |
| Target region | 2012 | 2013 | 2014 | 2015 | 2016 |
| East and Southeast Asia | 35.52 (10.49) | 42.36 (11.11) | 43.45 (13.73) | 62.21 (20.60) | 57.06 (15.65) |
| South and Central Asia | 78.13 (23.08) | 63.86 (16.75) | 83.65 (26.44) | 71.86 (23.80) | 88.02 (24.14) |
| Oceania | 10.97 (3.24) | 14.60 (3.83) | 9.35 (2.96) | 4.33 (1.44) | 7.15 (1.96) |
| Central Europe | 1.89 (0.56) | 2.50 (0.66) | 2.25 (0.71) | 5.51 (1.82) | 5.26 (1.44) |
| Americas | 19.05 (5.63) | 19.54 (5.13) | 25.07 (7.92) | 24.64 (8.16) | 37.75 (10.35) |
| Africa | 168.90 (49.90) | 214.49 (56.27) | 131.89 (41.69) | 112.64 (37.30) | 128.11 (35.14) |
| Middle East | 11.59 (3.42) | 5.00 (1.31) | 7.46 (2.36) | 7.07 (2.34) | 23.37 (6.41) |
| Multiple regions | 12.42 (3.67) | 18.85 (4.94) | 13.27 (4.19) | 13.68 (4.53) | 17.86 (4.90) |
| Total | 338.47 | 381.20 | 316.38 | 301.95 | 364.58 |
|  |  |  |  |  |  |
| C. multilateral |  |  |  |  |  |
| Target region | 2012 | 2013 | 2014 | 2015 | 2016 |
| East and Southeast Asia | 55.44 (11.88) | 35.77 (11.08) | 49.29 (10.48) | 30.23 (6.87) | 29.17 (6.71) |
| South and Central Asia | 100.51 (21.54) | 68.40 (21.18) | 73.58 (15.65) | 118.07 (26.85) | 115.88 (26.64) |
| Oceania | 5.50 (1.18) | 2.86 (0.89) | 3.84 (0.82) | 3.76 (0.86) | 3.88 (0.89) |
| Central Europe | 12.14 (2.60) | 7.29 (2.26) | 12.92 (2.75) | 9.34 (2.12) | 9.16 (2.11) |
| Americas | 20.37 (4.36) | 14.76 (4.57) | 18.86 (4.01) | 17.80 (4.05) | 15.41 (3.54) |
| Africa | 243.72 (52.22) | 169.35 (52.45) | 285.38 (60.68) | 240.57 (54.70) | 225.84 (51.91) |
| Middle East | 5.80 (1.24) | 4.23 (1.31) | 4.88 (1.04) | 3.00 (0.68) | 3.76 (0.87) |
| Multiple regions | 23.20 (4.97) | 20.23 (6.26) | 21.53 (4.58) | 17.01 (3.87) | 31.93 (7.34) |
| Total | 466.68 | 322.89 | 470.29 | 439.78 | 435.02 |
|  |  |  |  |  |  |
| D. total |  |  |  |  |  |
| Target region | 2012 | 2013 | 2014 | 2015 | 2016 |
| East and Southeast Asia | 100.35 (11.75) | 78.52 (10.93) | 93.28 (11.31) | 92.96 (10.65) | 104.24 (11.65) |
| South and Central Asia | 217.96 (25.53) | 145.94 (20.32) | 175.19 (21.24) | 208.76 (23.91) | 260.45 (29.11) |
| Oceania | 16.47 (1.93) | 17.46 (2.43) | 13.19 (1.60) | 8.10 (0.93) | 11.02 (1.23) |
| Central Europe | 14.03 (1.64) | 9.79 (1.36) | 32.99 (4.00) | 49.24 (5.64) | 15.02 (1.68) |
| Americas | 39.42 (4.62) | 34.30 (4.78) | 43.93 (5.33) | 42.43 (4.86) | 53.16 (5.94) |
| Africa | 412.62 (48.32) | 383.84 (53.45) | 417.27 (50.58) | 429.39 (49.18) | 372.48 (41.64) |
| Middle East | 17.39 (2.04) | 9.23 (1.28) | 14.30 (1.73) | 11.46 (1.31) | 28.41 (3.18) |
| Multiple regions | 35.62 (4.17) | 39.07 (5.44) | 34.80 (4.22) | 30.70 (3.52) | 49.79 (5.57) |
| Total | 853.87 | 718.16 | 824.95 | 873.04 | 894.57 |

**Supplementary table 2: Development assistance for health by channel, 2012–2016 (2016 USD in million, %)**

| Channel | 2012 | 2013 | 2014 | 2015 | 2016 |
| --- | --- | --- | --- | --- | --- |
| WHO | 44.12 (5.17) | 53.06 (7.39) | 65.17 (7.90) | 67.84 (7.77) | 98.53 (11.01) |
| UNAIDS | 2.59 (0.30) | 2.93 (0.41) | 4.17 (0.51) | 1.37 (0.16) | 1.05 (0.12) |
| UNFPA | 29.34 (3.44) | 24.20 (3.37) | 32.76 (3.97) | 36.34 (4.16) | 35.33 (3.95) |
| UNICEF | 78.58 (9.20) | 166.51 (23.19) | 87.10 (10.56) | 19.01 (2.18) | 50.14 (5.61) |
| UNDP | 2.43 (0.28) | 2.30 (0.32) | 1.41 (0.17) | 1.29 (0.15) | 1.16 (0.13) |
| Other UN agencies | 8.94 (1.05) | 8.31 (1.16) | 1.30 (0.16) | 1.29 (0.15) | 7.86 (0.88) |
| World Bank | 104.55 (12.24) | 100.79 (14.03) | 81.80 (9.92) | 140.66 (16.11) | 144.10 (16.11) |
| AfDB | 0.84 (0.10) | 1.01 (0.14) | 3.21 (0.39) | 2.26 (0.26) | 0.70 (0.08) |
| AsDB | 21.58 (2.53) | 14.31 (1.99) | 11.22 (1.36) | 6.55 (0.75) | 5.16 (0.58) |
| IADB | 0.51 (0.06) | 0.51 (0.07) | 1.27 (0.15) | 1.03 (0.12) | 0.37 (0.04) |
| Global Fund | 261.06 (30.57) | 114.21 (15.90) | 287.79 (34.89) | 212.15 (24.30) | 179.24 (20.04) |
| Gavi | 6.90 (0.81) | 8.47 (1.18) | 8.65 (1.05) | 19.57 (2.24) | 18.74 (2.10) |
| JICA | 233.45 (27.34) | 175.14 (24.39) | 188.62 (22.86) | 319.35 (36.58) | 315.33 (35.25) |
| Private-sector | – | – | – | – | 0.02 (0.00) |
| NGOs | 58.84 (6.89) | 44.19 (6.15) | 48.69 (5.90) | 43.86 (5.02) | 34.20 (3.82) |
| Others | 0.13 (0.02) | 2.22 (0.31) | 1.78 (0.22) | 0.46 (0.05) | 2.63 (0.29) |
| Total | 853.87 | 718.16 | 824.95 | 873.04 | 894.57 |

WHO: World Health Organization; UNAIDS: Joint United Nations Programme on HIV/AIDS; UNFPA: United Nations Population Fund; UNICEF: United Nations Children's Fund; UNDP: United Nations Development Programme; AfDB: African Development Bank; AsDB: Asian Development Bank; IADB: Inter-American Development Bank; Global Fund: The Global Fund to Fight AIDS, Tuberculosis and Malaria; Gavi: Gavi, The Vaccine Alliance; JICA: Japan International Cooperation Agency. Other UN agencies include Food and Agriculture Organization (FAO), United Nations Relief and Works Agency for Palestine Refugees in the Near East (UNRWA), World Food Programme (WFP), etc. NGOs include International Planned Parenthood Federation, etc. Others include Global Environment Facility (GEF), etc.

**Supplementary table 3: Developing assistance for health channeled through multilateral agencies, 2012–2016 (2016 USD in million, %)**

| Channel | Year | Earmarked funding/bi-multi (%) | Core funding (%) |
| --- | --- | --- | --- |
| WHO | 2012 | 1.52 (3.45) | 42.60 (96.55) |
|  | 2013 | 2.80 (5.28) | 50.26 (94.72) |
|  | 2014 | 19.55 (30.00) | 45.62 (70.00) |
|  | 2015 | 18.26 (26.91) | 49.58 (73.09) |
|  | 2016 | 18.60 (18.88) | 79.93 (81.12) |
| UNAIDS | 2012 | 0 (0) | 2.59 (100) |
|  | 2013 | 0 (0) | 2.93 (100) |
|  | 2014 | 0 (0) | 4.17 (100) |
|  | 2015 | 0 (0) | 1.37 (100) |
|  | 2016 | 0 (0) | 1.05 (100) |
| UNFPA | 2012 | 8.85 (30.17) | 20.49 (69.83) |
|  | 2013 | 0.93 (3.86) | 23.27 (96.14) |
|  | 2014 | 9.04 (27.60) | 23.71 (72.40) |
|  | 2015 | 16.17 (44.49) | 20.17 (55.51) |
|  | 2016 | 16.30 (46.15) | 19.02 (53.85) |
| UNICEF | 2012 | 76.70 (97.61) | 1.88 (2.39) |
|  | 2013 | 163.41 (98.14) | 3.10 (1.86) |
|  | 2014 | 87.10 (100) | 0 (0) |
|  | 2015 | 15.91 (83.69) | 3.10 (16.31) |
|  | 2016 | 47.43 (94.59) | 2.71 (5.41) |
| UNDP | 2012 | 0 (0) | 2.43 (100) |
|  | 2013 | 0 (0) | 2.30 (100) |
|  | 2014 | 0 (0) | 1.41 (100) |
|  | 2015 | 0 (0) | 1.29 (100) |
|  | 2016 | 0 (0) | 1.16 (100) |
| Other UN agencies | 2012 | 7.69 (86.01) | 1.25 (13.99) |
|  | 2013 | 6.70 (80.57) | 1.62 (19.43) |
|  | 2014 | 0 (0) | 1.30 (100) |
|  | 2015 | 0 (0) | 1.29 (100) |
|  | 2016 | 6.57 (83.62) | 1.29 (16.38) |
| World Bank | 2012 | 0 (0) | 104.55 (100) |
|  | 2013 | 0 (0) | 100.79 (100) |
|  | 2014 | 0 (0) | 81.80 (100) |
|  | 2015 | 0 (0) | 140.66 (100) |
|  | 2016 | 0 (0) | 144.10 (100) |
| African Development Bank | 2012 | 0 (0) | 0.84 (100) |
|  | 2013 | 0 (0) | 1.01 (100) |
|  | 2014 | 0 (0) | 3.21 (100) |
|  | 2015 | 0 (0) | 2.26 (100) |
|  | 2016 | 0 (0) | 0.70 (100) |
| Asian Development Bank | 2012 | 0 (0) | 21.58 (100) |
|  | 2013 | 0 (0) | 14.31 (100) |
|  | 2014 | 0 (0) | 11.22 (100) |
|  | 2015 | 0 (0) | 6.55 (100) |
|  | 2016 | 0 (0) | 5.16 (100) |
| Inter-American Development Bank | 2012 | 0 (0) | 0.51 (100) |
|  | 2013 | 0 (0) | 0.51 (100) |
|  | 2014 | 0 (0) | 1.27 (100) |
|  | 2015 | 0 (0) | 1.03 (100) |
|  | 2016 | 0 (0) | 0.37 (100) |
| Global Fund | 2012 | 0 (0) | 261.06 (100) |
|  | 2013 | 0 (0) | 114.21 (100) |
|  | 2014 | 0 (0) | 287.79 (100) |
|  | 2015 | 0 (0) | 212.15 (100) |
|  | 2016 | 0 (0) | 179.24 (100) |
| Gavi | 2012 | 0 (0) | 6.90 (100) |
|  | 2013 | 0 (0) | 8.47 (100) |
|  | 2014 | 0 (0) | 8.65 (100) |
|  | 2015 | 19.37 (98.96) | 0.20 (1.04) |
|  | 2016 | 18.58 (99.11) | 0.17 (0.89) |

WHO: World Health Organization; UNAIDS: Joint United Nations Programme on HIV/AIDS; UNFPA: United Nations Population Fund; UNICEF: United Nations Children's Fund; UNDP: United Nations Development Programme; AfDB: African Development Bank; AsDB: Asian Development Bank; IADB: Inter-American Development Bank; Global Fund: The Global Fund to Fight AIDS, Tuberculosis and Malaria; Gavi: Gavi, The Vaccine Alliance; JICA: Japan International Cooperation Agency. Other UN agencies include Food and Agriculture Organization (FAO), United Nations Relief and Works Agency for Palestine Refugees in the Near East (UNRWA), World Food Programme (WFP), etc.

**Supplementary table 4: Development assistance for health by health focus area, 2012–2016 (2016 USD in million, %): (A) bilateral (loans), (B) bilateral (grants), (C) multilateral, (D) total**

| A. bilateral (loans) |  |  |  |  |  |
| --- | --- | --- | --- | --- | --- |
| Health focus area | 2012 | 2013 | 2014 | 2015 | 2016 |
| Basic health care | – | – | – | – | 14.49 (15.26) |
| Basic health infrastructure | – | – | – | – | – |
| Basic nutrition | – | – | – | – | – |
| Health education | – | – | – | – | – |
| Health personnel development | – | – | – | – | – |
| Reproductive health care | – | – | – | – | – |
| Family planning | – | – | – | – | – |
| Infectious disease control | 31.84 (65.35) | 0.00 (0.03) | – | 76.18 (58.02) | 33.56 (35.34) |
| Malaria control | – | – | – | – | – |
| Tuberculosis control | – | – | – | – | – |
| STD control including HIV/AIDS | – | – | – | – | – |
| Health policy and administrative management | 9.38 (19.25) | – | – | – | 18.38 (19.36) |
| Medical education/training | – | – | – | – | – |
| Medical research | – | – | – | – | – |
| Medical services | 0.02 (0.04) | 2.80 (19.91) | 28.54 (74.56) | 43.16 (32.87) | 25.03 (26.36) |
| Population policy and administrative management | – | – | – | – | – |
| Personnel development for population and reproductive health | 7.48 (15.36) | 11.26 (80.05) | 9.74 (25.44) | 11.96 (9.11) | 3.50 (3.69) |
| Total | 48.72 | 14.07 | 38.28 | 131.31 | 94.97 |
|  |  |  |  |  |  |
| B. bilateral (grants) |  |  |  |  |  |
| Health focus area | 2012 | 2013 | 2014 | 2015 | 2016 |
| Basic health care | 17.70 (5.23) | 22.95 (6.02) | 10.41 (3.29) | 8.16 (2.70) | 13.59 (3.73) |
| Basic health infrastructure | 65.30 (19.29) | 28.94 (7.59) | 54.73 (17.30) | 66.97 (22.18) | 68.90 (18.90) |
| Basic nutrition | 32.96 (9.74) | 102.07 (26.78) | 56.46 (17.85) | 2.75 (0.91) | 0.30 (0.08) |
| Health education | 1.62 (0.48) | 3.75 (0.98) | 5.38 (1.70) | 3.22 (1.07) | 0.91 (0.25) |
| Health personnel development | 4.22 (1.25) | 10.76 (2.82) | 1.01 (0.32) | 8.12 (2.69) | 19.22 (5.27) |
| Reproductive health care | 32.99 (9.75) | 39.65 (10.40) | 46.08 (14.56) | 45.95 (15.22) | 44.67 (12.25) |
| Family planning | 7.63 (2.25) | 0.09 (0.02) | – | – | – |
| Infectious disease control | 54.65 (16.15) | 21.74 (5.70) | 38.55 (12.18) | 46.84 (15.51) | 65.68 (18.01) |
| Malaria control | 1.92 (0.57) | 28.75 (7.54) | 1.53 (0.48) | 3.25 (1.08) | 1.91 (0.52) |
| Tuberculosis control | 6.93 (2.05) | 3.57 (0.94) | 13.75 (4.35) | 3.75 (1.24) | 2.96 (0.81) |
| STD control including HIV/AIDS | 10.51 (3.10) | 8.88 (2.33) | 6.52 (2.06) | 4.03 (1.33) | 0.96 (0.26) |
| Health policy and administrative management | 68.30 (20.18) | 53.55 (14.05) | 44.63 (14.11) | 61.46 (20.35) | 73.82 (20.25) |
| Medical education/training | 0.34 (0.10) | 1.01 (0.27) | 6.80 (2.15) | 0.34 (0.11) | 5.07 (1.39) |
| Medical research | 0.16 (0.05) | 6.73 (1.76) | 0.31 (0.10) | – | – |
| Medical services | 24.10 (7.12) | 47.27 (12.40) | 30.23 (9.55) | 46.82 (15.51) | 66.60 (18.27) |
| Population policy and administrative management | 8.48 (2.51) | 0.93 (0.25) | – | – | – |
| Personnel development for population and reproductive health | 0.65 (0.19) | 0.54 (0.14) | – | 0.30 (0.10) | – |
| Total | 338.47 | 381.20 | 316.38 | 301.95 | 364.58 |
|  |  |  |  |  |  |
| C. multilateral |  |  |  |  |  |
| Health focus area | 2012 | 2013 | 2014 | 2015 | 2016 |
| Basic health care | 38.83 (8.32) | 42.58 (13.19) | 34.91 (7.42) | 56.19 (12.78) | 58.39 (13.42) |
| Basic health infrastructure | 3.04 (0.65) | 6.45 (2.00) | 4.15 (0.88) | 4.76 (1.08) | 4.82 (1.11) |
| Basic nutrition | 9.25 (1.98) | 9.14 (2.83) | 9.54 (2.03) | 13.32 (3.03) | 20.91 (4.81) |
| Health education | 1.61 (0.34) | 2.24 (0.69) | 1.18 (0.25) | 1.84 (0.42) | 1.56 (0.36) |
| Health personnel development | 2.97 (0.64) | 1.65 (0.51) | 3.08 (0.65) | 4.41 (1.00) | 4.56 (1.05) |
| Reproductive health care | 11.67 (2.50) | 12.54 (3.88) | 10.59 (2.25) | 11.50 (2.61) | 24.20 (5.56) |
| Family planning | 1.50 (0.32) | 2.02 (0.62) | 2.55 (0.54) | 2.49 (0.57) | 4.86 (1.12) |
| Infectious disease control | 21.31 (4.57) | 27.72 (8.58) | 15.64 (3.33) | 66.34 (15.09) | 31.71 (7.29) |
| Malaria control | 86.15 (18.46) | 36.33 (11.25) | 89.65 (19.06) | 63.62 (14.47) | 58.68 (13.49) |
| Tuberculosis control | 40.77 (8.74) | 24.76 (7.67) | 45.62 (9.70) | 33.67 (7.66) | 35.45 (8.15) |
| STD control including HIV/AIDS | 160.75 (34.45) | 76.62 (23.73) | 167.87 (35.69) | 106.93 (24.32) | 85.70 (19.70) |
| Health policy and administrative management | 64.90 (13.91) | 57.14 (17.70) | 57.03 (12.13) | 41.22 (9.37) | 71.50 (16.44) |
| Medical education/training | 3.27 (0.70) | 2.25 (0.70) | 2.31 (0.49) | 1.86 (0.42) | 3.58 (0.82) |
| Medical research | 0.17 (0.04) | 0.21 (0.07) | 2.88 (0.61) | 3.18 (0.72) | 5.44 (1.25) |
| Medical services | 6.51 (1.40) | 5.33 (1.65) | 5.37 (1.14) | 8.35 (1.90) | 10.79 (2.48) |
| Population policy and administrative management | 13.97 (2.99) | 15.78 (4.89) | 17.66 (3.76) | 16.30 (3.71) | 8.88 (2.04) |
| Personnel development for population and reproductive health | – | 0.13 (0.04) | 0.24 (0.05) | 3.80 (0.86) | 4.00 (0.92) |
| Total | 466.68 | 322.89 | 470.29 | 439.78 | 435.02 |
|  |  |  |  |  |  |
| D. total |  |  |  |  |  |
| Health focus area | 2012 | 2013 | 2014 | 2015 | 2016 |
| Basic health care | 56.53 (6.62) | 65.53 (9.12) | 45.32 (5.49) | 64.35 (7.37) | 86.47 (9.67) |
| Basic health infrastructure | 68.33 (8.00) | 35.39 (4.93) | 58.89 (7.14) | 71.73 (8.22) | 73.72 (8.24) |
| Basic nutrition | 42.21 (4.94) | 111.21 (15.49) | 66.01 (8.00) | 16.07 (1.84) | 21.20 (2.37) |
| Health education | 3.23 (0.38) | 6.00 (0.83) | 6.56 (0.80) | 5.06 (0.58) | 2.47 (0.28) |
| Health personnel development | 7.19 (0.84) | 12.41 (1.73) | 4.09 (0.50) | 12.53 (1.44) | 23.77 (2.66) |
| Reproductive health care | 44.66 (5.23) | 52.19 (7.27) | 56.67 (6.87) | 57.44 (6.58) | 68.86 (7.70) |
| Family planning | 9.13 (1.07) | 2.11 (0.29) | 2.55 (0.31) | 2.49 (0.28) | 4.86 (0.54) |
| Infectious disease control | 107.80 (12.62) | 49.47 (6.89) | 54.19 (6.57) | 189.36 (21.69) | 130.95 (14.64) |
| Malaria control | 88.08 (10.32) | 65.08 (9.06) | 91.18 (11.05) | 66.88 (7.66) | 60.58 (6.77) |
| Tuberculosis control | 47.70 (5.59) | 28.33 (3.94) | 59.37 (7.20) | 37.41 (4.29) | 38.42 (4.29) |
| STD control including HIV/AIDS | 171.26 (20.06) | 85.51 (11.91) | 174.39 (21.14) | 110.96 (12.71) | 86.66 (9.69) |
| Health policy and administrative management | 142.58 (16.70) | 110.69 (15.41) | 101.66 (12.32) | 102.68 (11.76) | 163.70 (18.30) |
| Medical education/training | 3.62 (0.42) | 3.26 (0.45) | 9.11 (1.10) | 2.20 (0.25) | 8.65 (0.97) |
| Medical research | 0.33 (0.04) | 6.94 (0.97) | 3.19 (0.39) | 3.18 (0.36) | 5.44 (0.61) |
| Medical services | 30.63 (3.59) | 55.40 (7.71) | 64.14 (7.77) | 98.34 (11.26) | 102.43 (11.45) |
| Population policy and administrative management | 22.45 (2.63) | 16.72 (2.33) | 17.66 (2.14) | 16.30 (1.87) | 8.88 (0.99) |
| Personnel development for population and reproductive health | 8.13 (0.95) | 11.93 (1.66) | 9.98 (1.21) | 16.06 (1.84) | 7.50 (0.84) |
| Total | 853.87 | 718.16 | 824.95 | 873.04 | 894.57 |

STD: sexually transmitted disease. CRS purpose code: Basic health care = 12220; Basic health infrastructure = 12230; Basic nutrition = 12240; Health education = 12261; Health personnel development = 12281; Reproductive health care = 13020; Family planning = 13030; Infectious disease control = 12250; Malaria control = 12262; Tuberculosis control = 12263; STD control including HIV/AIDS = 13040; Health policy and administrative management = 12110; Medical education/training = 12181; Medical research=12182; Medical services = 12191; Population policy and administrative management = 13010; Personnel development for population and reproductive health = 13081.

**Supplementary table 5: Development assistance for health for primary healthcare and health system strengthening, 2012–2016 (2016 USD in million, %): (A) bilateral (loans), (B) bilateral (grants), (C) multilateral, (D) total**

| A. bilateral (loans) |  |  |  |  |  |
| --- | --- | --- | --- | --- | --- |
|  | 2012 | 2013 | 2014 | 2015 | 2016 |
| PHC |  |  |  |  |  |
| PHC definition #1 | – | – | – | – | 14.49 (15.26) |
| PHC definition #2 | – | – | – | – | – |
| PHC definition #3 | 31.84 (65.35) | 0.00 (0.03) | – | 76.18 (58.02) | 33.56 (35.34) |
| PHC definition #4 | – | – | – | – | – |
| Broader PHC definition | 31.84 (65.35) | 0.00 (0.03) | – | 76.18 (58.02) | 48.05 (50.60) |
| HSS |  |  |  |  |  |
| HSS definition #1 | 9.38 (19.25) | – | – | – | 18.38 (19.36) |
| HSS definition #2 | 0.02 (0.04) | 2.80 (19.91) | 28.54 (74.56) | 43.16 (32.87) | 25.03 (26.36) |
| HSS definition #3 | 7.48 (15.36) | 11.26 (80.05) | 9.74 (25.44) | 11.96 (9.11) | 3.50 (3.69) |
| Broader HSS definition | 16.88 (34.65) | 14.07 (99.97) | 38.28 (100.00) | 55.13 (41.98) | 46.92 (49.40) |
|  |  |  |  |  |  |
| B. bilateral (grants) |  |  |  |  |  |
|  | 2012 | 2013 | 2014 | 2015 | 2016 |
| PHC |  |  |  |  |  |
| PHC definition #1 | 121.79 (35.98) | 168.48 (44.20) | 127.99 (40.46) | 89.21 (29.55) | 102.91 (28.23) |
| PHC definition #2 | 40.63 (12.00) | 39.74 (10.42) | 46.08 (14.56) | 45.95 (15.22) | 44.67 (12.25) |
| PHC definition #3 | 63.50 (18.76) | 54.07 (14.18) | 53.83 (17.01) | 53.83 (17.83) | 70.55 (19.35) |
| PHC definition #4 | 10.51 (3.10) | 8.88 (2.33) | 6.52 (2.06) | 4.03 (1.33) | 0.96 (0.26) |
| Broader PHC definition | 236.43 (69.85) | 271.17 (71.14) | 234.42 (74.09) | 193.03 (63.93) | 219.09 (60.09) |
| HSS |  |  |  |  |  |
| HSS definition #1 | 68.30 (20.18) | 53.55 (14.05) | 44.63 (14.11) | 61.46 (20.35) | 73.82 (20.25) |
| HSS definition #2 | 24.61 (7.27) | 55.01 (14.43) | 37.34 (11.80) | 47.16 (15.62) | 71.67 (19.66) |
| HSS definition #3 | 9.13 (2.70) | 1.47 (0.39) | 0.00 (0.00) | 0.30 (0.10) | – |
| Broader HSS definition | 102.04 (30.15) | 110.02 (28.86) | 81.97 (25.91) | 108.92 (36.07) | 145.49 (39.91) |
|  |  |  |  |  |  |
| C. multilateral |  |  |  |  |  |
|  | 2012 | 2013 | 2014 | 2015 | 2016 |
| PHC |  |  |  |  |  |
| PHC definition #1 | 55.70 (11.94) | 62.06 (19.22) | 52.87 (11.24) | 80.52 (18.31) | 90.23 (20.74) |
| PHC definition #2 | 13.17 (2.82) | 14.56 (4.51) | 13.14 (2.79) | 13.98 (3.18) | 29.06 (6.68) |
| PHC definition #3 | 148.23 (31.76) | 88.80 (27.50) | 150.91 (32.09) | 163.64 (37.21) | 125.84 (28.93) |
| PHC definition #4 | 160.75 (34.45) | 76.62 (23.73) | 167.87 (35.69) | 106.93 (24.32) | 85.70 (19.70) |
| Broader PHC definition | 377.86 (80.97) | 242.04 (74.96) | 384.79 (81.82) | 365.07 (83.01) | 330.83 (76.05) |
| HSS |  |  |  |  |  |
| HSS definition #1 | 64.90 (13.91) | 57.14 (17.70) | 57.03 (12.13) | 41.22 (9.37) | 71.50 (16.44) |
| HSS definition #2 | 9.95 (2.13) | 7.80 (2.41) | 10.56 (2.25) | 13.39 (3.05) | 19.81 (4.55) |
| HSS definition #3 | 13.97 (2.99) | 15.91 (4.93) | 17.91 (3.81) | 20.10 (4.57) | 12.88 (2.96) |
| Broader HSS definition | 88.82 (19.03) | 80.85 (25.04) | 85.50 (18.18) | 74.71 (16.99) | 104.19 (23.95) |
|  |  |  |  |  |  |
| D. total |  |  |  |  |  |
|  | 2012 | 2013 | 2014 | 2015 | 2016 |
| PHC |  |  |  |  |  |
| PHC definition #1 | 177.49 (20.79) | 230.54 (32.10) | 180.86 (21.92) | 169.73 (19.44) | 207.64 (23.21) |
| PHC definition #2 | 53.80 (6.30) | 54.30 (7.56) | 59.22 (7.18) | 59.93 (6.86) | 73.72 (8.24) |
| PHC definition #3 | 243.57 (28.53) | 142.87 (19.89) | 204.74 (24.82) | 293.65 (33.64) | 229.95 (25.70) |
| PHC definition #4 | 171.26 (20.06) | 85.51 (11.91) | 174.39 (21.14) | 110.96 (12.71) | 86.66 (9.69) |
| Broader PHC definition | 646.12 (75.67) | 513.22 (71.46) | 619.21 (75.06) | 634.28 (72.65) | 597.97 (66.84) |
| HSS |  |  |  |  |  |
| HSS definition #1 | 142.58 (16.70) | 110.69 (15.41) | 101.66 (12.32) | 102.68 (11.76) | 163.70 (18.30) |
| HSS definition #2 | 34.58 (4.05) | 65.61 (9.14) | 76.44 (9.27) | 103.72 (11.88) | 116.52 (13.03) |
| HSS definition #3 | 30.59 (3.58) | 28.65 (3.99) | 27.65 (3.35) | 32.36 (3.71) | 16.38 (1.83) |
| Broader HSS definition | 207.75 (24.33) | 204.94 (28.54) | 205.74 (24.94) | 238.76 (27.35) | 296.60 (33.16) |

PHC: public health care; HSS: health system strengthening. PHC definition #1 = Basic health care and infrastructure (CRS purpose codes: 12220, 12230, 12240, 12261, 12281); PHC definition #2 = Reproductive health care and family planning (13020, 13030); PHC definition #3 = Infectious disease control, including malaria and tuberculosis (12250, 12262, 12263); PHC definition #4 = Sexually transmitted disease (STD) control including HIV/AIDS (13040); Broader PHC definition=Definition #1 + #2 + #3 + #4; HSS definition #1 = Health policy, administration & management (12110); HSS definition #2 = Medical services, training & research (12181, 12182, 12191); HSS definition #3 = Population policy & administration (13010, 13081); Broader HSS definition = Definition #1 + #2 + #3.
